# Recognizing Starvation Ketoacidosis in Acute Care Surgery: A Case Report and Literature Review

**DOI:** 10.7759/cureus.106809

**Published:** 2026-04-10

**Authors:** Truman Archer, Sawyer Archer, David S Harper, Ahmed Elfadaly, Mohamed Elfedaly

**Affiliations:** 1 Department of Surgery, Texas Tech University Health Sciences Center, Amarillo, USA; 2 Department of Surgery, San Joaquin General Hospital, French Camp, USA

**Keywords:** acute care surgery and trauma, high anion gap metabolic acidosis, non-diabetic ketoacidosis, small bowel perforation, starvation ketoacidosis

## Abstract

Starvation ketoacidosis is an underrecognized metabolic complication in non-diabetic surgical patients who experience prolonged fasting. While commonly associated with diabetic ketoacidosis, high anion gap metabolic acidosis can occur in patients without diabetes following several days of inadequate oral intake, particularly when combined with surgical stress.

A 71-year-old Hispanic woman with a history of open ventral hernia repair in Mexico six weeks prior presented to the emergency department with abdominal pain following a fall. Computed tomography revealed concern for small bowel perforation. She underwent emergent exploratory laparotomy with abdominal washout, small bowel resection (10 cm distal jejunum), and temporary wound vacuum-assisted closure, followed by re-exploratory laparotomy with small bowel anastomosis and abdominal closure the following day. On postoperative day 3, the patient developed respiratory distress and altered mental status. Arterial blood gas analysis revealed severe metabolic acidosis with pH 7.045, pCO2 12.7 mmHg, HCO3 11.0 mEq/L, and base excess -25.1 mEq/L. Lactate was normal. Given the clinical context of prolonged poor oral intake and the severity of acidosis disproportionate to the lactate level despite lack of a diagnosis of diabetes, serum ketone bodies were measured and found to be markedly elevated at 6.8 mmol/L. The patient was diagnosed with starvation ketoacidosis and, given the patient’s inability to tolerate enteral intake and ongoing postoperative status, was immediately treated with total parenteral nutrition and intravenous sodium bicarbonate to provide continuous carbohydrate delivery. Within several hours, the anion gap normalized, and the patient's clinical status improved dramatically.

This case emphasizes the importance of maintaining a high index of suspicion for starvation ketoacidosis in acute care surgery patients presenting after prolonged periods of inadequate oral intake, particularly those with complex surgical histories requiring multiple operations.

## Introduction

Ketoacidosis is most commonly associated with diabetic ketoacidosis; however, it can also arise from starvation and alcoholic states, which are frequently underrecognized in clinical practice [1]. Starvation ketoacidosis arises during prolonged caloric deprivation when metabolism shifts away from carbohydrate utilization toward lipid oxidation, leading to increased ketone body production [2]. While this is typically a protective mechanism permitting survival during periods of nutrient deprivation, prolonged ketogenesis can lead to ketoacidosis, a potentially life-threatening metabolic disorder [1]. Starvation ketoacidosis is typically mild due to residual insulin activity; however, superimposed surgical stress and catecholamine surge may markedly amplify ketogenesis.

In surgical patients, the risk of starvation ketoacidosis may be amplified by several factors. Patients presenting with acute abdominal pathology often experience several days of poor oral intake due to nausea, vomiting, and abdominal pain prior to seeking medical attention. The additional stress of surgery and general anesthesia further exacerbates metabolic derangements [3,4]. Despite the clinical significance of this condition, starvation ketoacidosis remains underrecognized in the acute care surgery setting, leading to potential delays in diagnosis and treatment [1,5].

Laboratory values in starvation ketoacidosis are typically significant for signs of an elevated anion gap, such as low serum bicarbonate, accompanied by normal glucose levels, unlike diabetic ketoacidosis. Notably, markers of other common sources of acidosis, such as an elevated serum lactate or creatinine, are absent. However, clinical features are nonspecific and similar to other causes of high anion-gap metabolic acidosis, such as nausea, altered mental status, and tachypnea. The mainstay of treatment is glucose administration to restore metabolic balance. Starvation ketoacidosis should be in the differential for any postoperative patient, even those without diabetes, with an unexplained high anion gap metabolic acidosis.

We present a case of severe starvation ketoacidosis in a non-diabetic patient following emergency small bowel resection for small bowel perforation, emphasizing the importance of early recognition and targeted management in critically ill surgical patients.

## Case presentation

A 71-year-old Hispanic woman presented to the emergency department with abdominal pain following a fall. Her past surgical history was significant for open ventral hernia repair performed in Mexico approximately six weeks prior to presentation. She had no history of diabetes mellitus or other significant medical comorbidities. 

**Figure 1:**
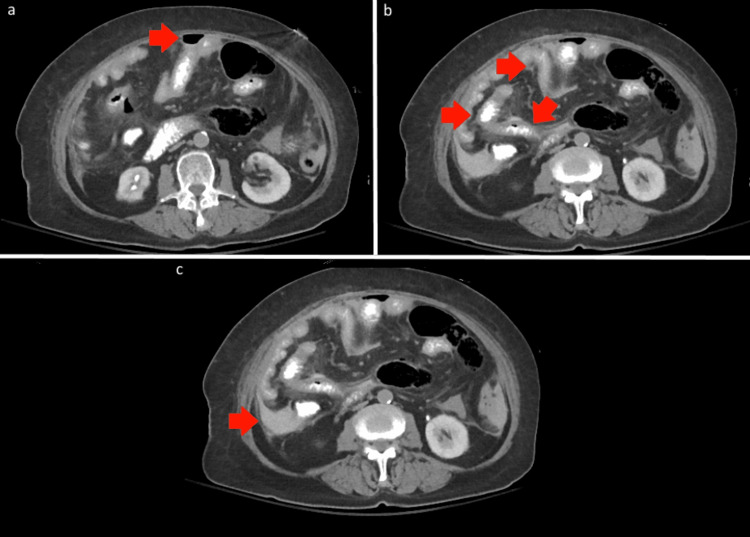
Computed tomography after initial patient presentation showing (a) intraperitoneal free air, (b) small bowel wall thickening, and (c) intraperitoneal fluid collection

On presentation, computed tomography of the abdomen and pelvis revealed findings concerning for small bowel perforation including intraperitoneal free air, small bowel inflammation, and fluid collections. These findings are shown in Figure 1. Initial laboratory evaluation showed white blood cell count of 7.7 × 10³/μL (reference: 4.0-10.6 x 10³/μL) and lactic acid of 4.0 mmol/L (reference: 0.4-2.0 mmol/L). General surgery was consulted, and given the acute nature of the presentation and concern for bowel perforation, the patient was taken emergently to the operating room

Small bowel perforation was confirmed during exploratory laparotomy. She underwent abdominal washout, small bowel resection (10 cm of distal jejunum), debridement of the previous ventral hernia wound (4 × 4 cm), and vacuum-assisted closure of the wound. The following day, she returned to the operating room for re-exploratory laparotomy, small bowel anastomosis, abdominal closure, and vacuum-assisted wound closure. The patient was transferred to the surgical intensive care unit for postoperative monitoring. The initially elevated lactate normalized following resuscitation, reducing concern for ongoing tissue hypoperfusion.

On postoperative day 3, the patient developed worsening dyspnea and respiratory distress accompanied by altered mental status. Arterial blood gas analysis and laboratory values revealed severe metabolic acidosis with appropriate respiratory compensation confirmed by Kussmaul respirations on physical exam, as demonstrated in Table 1. However, the patient’s lactate was normal, supporting an alternative to usual causes, such as hypoperfusion, of severe acidosis (anion gap of 20 mmol/L and base excess of -25.1 mEq/L). Blood glucose was within normal limits, and renal function was preserved. Significantly, the patient had no history of diabetes mellitus. 

**Table 1:** Patient’s laboratory values showing evidence of high anion gap metabolic acidosis

Lab	Value	Reference
pH	7.045	7.35 to 7.45
pCO_2_	12.7 mmHg	35 to 45 mmHg
pO_2_	86 mmHg	80 to 100 mmHg
HCO_3_^-^	11.0 mEq/L	22 to 26 mEq/L
Base Excess	-25.1 mEq/L	-2 to +2 mEq/L
Anion Gap	20 mmol/L	9 to 18 mmol/L
Lactate	0.60 mmol/L	0.4 - 2.0 mmol/L
Oxygen Saturation	95% on 2/L/min Nasal Cannula	94 to 100%

In summary, the patient's acute presentation on postoperative day 3 was characterized by respiratory distress with tachypnea and altered mental status, while subsequent laboratory values were significant for a low pH and elevated anion gap of 20 mmol/L. Glucose levels remained in the 80-120 mg/dL range and lactate level was within normal limits at 0.60 mmol/L. Other causes of elevated anion gap, such as toxin or drug ingestion, were not consistent with the patient's history because she remained in the hospital setting in the days between surgery and development of acidosis. Given the patient's history of prolonged poor oral intake surrounding her acute presentation and multiple surgical procedures, and the absence of other identifiable causes of severe metabolic acidosis, serum ketone bodies were measured. Beta-hydroxybutyrate was markedly elevated at 6.8 mmol/L (reference: <0.6 mmol/L), confirming the diagnosis of starvation ketoacidosis. 

Given the patient’s inability to tolerate enteral intake and ongoing postoperative status, parenteral nutrition was initiated to provide continuous carbohydrate substrate and suppress ketogenesis, along with intravenous sodium bicarbonate to address severe acidemia. The patient demonstrated rapid clinical improvement, with normalization of the anion gap within several hours. Her respiratory distress resolved, mental status improved, and supplemental oxygen was successfully weaned.

The patient's subsequent hospital course was complicated by oral thrush (treated with nystatin), poor appetite requiring continued nutritional support, and respiratory infection with sputum culture positive for ESBL-producing Escherichia coli (treated with meropenem for six days). She was eventually transitioned to oral intake with the assistance of speech-language pathology and dietitian services. The patient was discharged home in stable condition after a 29-day hospitalization.

## Discussion

This case illustrates the presentation, diagnosis, and management of starvation ketoacidosis in a non-diabetic patient following major abdominal surgery. During prolonged fasting, reduced circulating insulin in combination with elevations in counter-regulatory hormones, such as glucagon and cortisol, drives adipose tissue lipolysis and subsequent hepatic ketone production [1,2]. Although ketone bodies serve as an alternative energy substrate during periods of caloric deprivation, excessive accumulation results in metabolic acidosis [6]. In prolonged fasting states, beta-hydroxybutyrate levels may reach 2.5-4.5 mmol/L after 5-7 days in healthy individuals [2]. The markedly elevated level observed in this patient (6.8 mmol/L) reflects severe metabolic derangement, likely potentiated by surgical stress and repeated operative interventions. The surgical stress response further exacerbates metabolic derangements through catecholamine and cortisol-mediated catabolism, promoting lipolysis and ketogenesis [3,4]. In this patient, the combination of recent surgery, acute bowel perforation, and two major operations within 48 hours created a profoundly catabolic state, predisposing to severe ketoacidosis.

Similarly, the arterial blood gas findings in this case were particularly striking. The degree of acidemia (pH 7.045) and base deficit (−25.1 mEq/L) indicated profound metabolic acidosis. The pCO2 of 12.7 mmHg demonstrates maximal respiratory compensation through hyperventilation (Kussmaul respirations). The discrepancy between the severity of high anion gap metabolic acidosis and a normal lactate level in the setting of intact kidney function was a key clinical clue that prompted investigation for alternative causes, ultimately leading to the measurement of serum beta-hydroxybutyrate.

Prior reports have described starvation ketoacidosis in surgical patients. Zhou and Luo reported severe metabolic acidosis following prolonged preoperative fasting in a patient undergoing laparoscopic hysterectomy, successfully treated with glucose and sodium bicarbonate [3]. Similarly, Song and Cao described starvation ketoacidosis after bariatric surgery, initially misdiagnosed as diabetic ketoacidosis but rapidly responsive to glucose administration [7]. These cases and the case reported here underscore the importance of including starvation ketoacidosis in the differential diagnosis of unexplained high anion gap metabolic acidosis in surgical populations.

The treatment of starvation ketoacidosis differs fundamentally from diabetic ketoacidosis. While diabetic ketoacidosis requires insulin administration to suppress ketogenesis, starvation ketoacidosis is treated primarily with glucose supplementation [3,7]. The provision of exogenous glucose stimulates endogenous insulin release, which suppresses lipolysis and ketone production while promoting ketone body utilization [7]. Routine use of sodium bicarbonate is controversial, but it may be administered in the case of severe acidosis (pH <7.1) [3]. Given our patient's pH of 7.045, sodium bicarbonate administration was appropriate. The combination of total parenteral nutrition and bicarbonate resulted in rapid resolution of acidosis within hours, a response time consistent with other reported cases [3,7].

The rapid clinical improvement following treatment is characteristic of starvation ketoacidosis and helps confirm the diagnosis. Unlike diabetic ketoacidosis, which may require prolonged insulin infusion and intensive care management, starvation ketoacidosis typically resolves quickly once glucose is provided [7]. This dramatic response was evident in our patient, whose anion gap normalized and clinical status improved within several hours of treatment initiation.

This case has several important implications for perioperative and critical care management. First, it highlights the need for increased awareness of starvation ketoacidosis in patients with prolonged fasting, particularly in the setting of acute surgical illness. Second, it emphasizes the importance of measuring serum ketones in patients with unexplained high anion gap metabolic acidosis, even in the absence of diabetes, especially when acidosis severity is disproportionate to lactate levels [1,5]. Third, it demonstrates that prompt recognition and appropriate treatment can result in rapid reversal and favorable outcomes. Emerging literature has also highlighted the metabolic consequences of prolonged perioperative fasting [8-10]. Even short fasting durations may result in ketosis and metabolic acidosis [8]. Enhanced Recovery After Surgery (ERAS) protocols advocate minimizing fasting duration and incorporating preoperative carbohydrate loading to mitigate metabolic stress [3,9,10]. Although prolonged fasting was unavoidable in this patient due to emergent pathology, this case reinforces the importance of early nutritional support in critically ill surgical patients.

## Conclusions

Starvation ketoacidosis represents an important and frequently underrecognized cause of high anion gap metabolic acidosis in non-diabetic surgical patients, particularly in the setting of prolonged fasting and perioperative physiologic stress. This case demonstrates that patients presenting with acute abdominal pathology requiring emergency surgery are at heightened risk, especially when subjected to the additional stress of multiple operations, due to the combined effects of inadequate nutritional intake and a sustained catabolic state. Clinicians should maintain a high index of suspicion for starvation ketoacidosis in patients with unexplained severe metabolic acidosis, normal glucose, and a history of prolonged fasting, particularly when the degree of acidosis exceeds what would be expected from the lactate level alone. Measurement of serum ketone bodies is essential for diagnosis, and prompt treatment with glucose supplementation can lead to rapid resolution of this potentially life-threatening condition. Unlike other causes of ketoacidosis, treatment is centered on carbohydrate repletion, which can result in rapid reversal of metabolic derangements and clinical improvement. Greater awareness of this metabolic crisis among critical care and acute care surgery teams may reduce diagnostic delays and improve patient outcomes.

## References

[1] Bashir B, Fahmy AA, Raza F, Banerjee M (2021). Non-diabetic ketoacidosis: a case series and literature review. Postgrad Med J.

[2] Grey NJ, Karl I, Kipnis DM (1975). Physiologic mechanisms in the development of starvation ketosis in man. Diabetes.

[3] Zhou W, Luo L (2019). Preoperative prolonged fasting causes severe metabolic acidosis: a case report. Medicine (Baltimore).

[4] Wei KY, Chang SY, Wang SH, Su HY, Tsai CL (2016). Short-term starvation with a near-fatal asthma attack induced ketoacidosis in a nondiabetic pregnant woman: a case report. Medicine (Baltimore).

[5] Gall AJ, Duncan R, Badshah A (2020). Starvation ketoacidosis on the acute medical take. Clin Med (Lond).

[6] Kamel KS, Halperin ML (2015). Acid-base problems in diabetic ketoacidosis. N Engl J Med.

[7] Song R, Cao S (2018). Post-bariatric surgery starvation ketoacidosis and lipase elevation in the absence of DKA or pancreatitis. Am J Emerg Med.

[8] Lim L, Park SJ, Kang C, Oh SY, Ryu HG, Lee H (2024). Perioperative urinary ketosis and metabolic acidosis in patients fasted for undergoing gynecologic surgery. Acta Anaesthesiol Scand.

[9] Pimenta GP, de Aguilar-Nascimento JE (2014). Prolonged preoperative fasting in elective surgical patients: why should we reduce it?. Nutr Clin Pract.

[10] Awad S, Lobo DN (2012). Metabolic conditioning to attenuate the adverse effects of perioperative fasting and improve patient outcomes. Curr Opin Clin Nutr Metab Care.

